# Antioxidant and antihyperlipidemic effect of *Solanum nigrum* fruit extract on the experimental model against chronic ethanol toxicity

**DOI:** 10.4103/0973-1296.59965

**Published:** 2010-02-13

**Authors:** Vadivel Arulmozhi, Mani Krishnaveni, Kandhan Karthishwaran, Ganesan Dhamodharan, Sankaran Mirunalini

**Affiliations:** *Department of Biochemistry and Biotechnology, Annamalai University, Annamalainagar - 608 002, Tamil Nadu, India*

**Keywords:** Antihyperlipidemic, antioxidant, ethanol, *Solanum nigrum*

## Abstract

The possible protective effect of *Solanum nigrum* fruit extract (SNFEt) was investigated for its antioxidant and antihyperlipidemic activity against ethanol-induced toxicity in rats. The experimental animals were intoxicated with 20% ethanol (7.9 g/kg/day) for 30 days via gastric intubation. SNFEt was administered at the dose of 250 mg/kg body weight along with the daily dose of ethanol for 30 days. From the result it was observed that ethanol-induced rats showed a significant elevation in the levels of Thiobarbituric acid reactive substances (TBARS), which lowered the antioxidant defense systems, such as, reduced glutathione (GSH) and vitamins C and E, when compared to the controls. In the lipid profiles, the levels of total cholesterol (TC), triglycerides (TG), low density lipoproteins (LDL), very low density lipoproteins (VLDL), free fatty acids (FFA), and phospholipids were significantly elevated in the ethanol-induced group, whereas, the high density lipoproteins (HDL) were found to be reduced in the plasma, and the phospholipid levels were significantly decreased in the tissues. Supplementation of SNFEt improved the antioxidant status by decreasing the levels of TBARS and altering the lipid profiles to near normal. These activities were also compared to the standard drug silymarin (25 mg/kg body weight). Thus the findings of the present study indicated a significant antioxidant and antihyperlipidemic activity of *Solanum nigrum* fruits, which offered protection against ethanol-induced toxicity.

## INTRODUCTION

Ethanol, a principal psychoactive constituent in alcoholic beverages is one of the most abused drugs worldwide. Nowadays, it has become a growing medical, Social and economic issue faced by adults and the younger generation. Moderate drinking may not have any significant health problems, however, chronic heavy drinking causes a variety of liver problems including excess fat in the liver (fatty liver), alcoholic hepatitis (inflammation in the liver) and cirrhosis (permanent scarring of the liver).[1]

Pathogenesis of alcoholic liver disease is mainly due to the generation of an excessive amount of reactive oxygen species (ROS), resulting in the detrimental effects of the cellular antioxidant defense system,[2,3] as well as, enhancement of the lipid peroxidation process.[4] Moreover, an increased intake of ethanol is known to increase the levels of lipids, which lead to hyperlipidemia.[5] Thus, chronic and excess alcohol consumption may accelerate an oxidative mechanism directly or indirectly, which eventually produces cell death and tissue damage.[6] Although significant progress has been made in understanding the pathogenesis of alcoholic liver disease, treatment options are limited as well as problematic. Therefore, alternative treatments for liver disorders are needed to replace the existing synthetic drugs.

Antioxidants of plant origin have been reported to either inhibit or prevent the development of fundamental cellular disturbances resulting from excessive alcohol consumption.[7] *Solanum nigrum* (family: Solanaceae) commonly known as black nightshade, grows as a weed, found in the dry parts of India and other parts of the world. It has a long history of medicinal usage and has been used as a traditional folk medicine for treating various ailments such as pain, inflammation, fever and liver disorders.[8,9] Generally, black nightshade is very rich in nutritive values, which are capable of supplying minerals, vitamins, proteins, and certain hormone precursors.[10] This herb elaborates a wide spectrum of medicinal properties such as anticancer,[11] antioxidant,[12] neuroprotective,[13] cytoprotective,[14] antiulcer,[15] antimicrobial,[16] antinociceptive and antipyretic properties.[17] It has been claimed that *Solanum nigrum* fruits in particular are an excellent remedy for liver disorders.[18] It also has the capacity to scavenge hydroxyl radicals[14] by inhibiting oxidative damage.[19] A recent report has shown that *Solanum nigrum* exerts protection against liver fibrosis.[20] In India *Solanum nigrum* has been chosen as the important ingredient for herbal formulations, namely Liv 52, which is mainly used for treating liver diseases.[21] Several photochemicals have been identified and isolated from *Solanum nigrum*, which contain alkaloids, flavonoids, saponins, tannins, phytic acid and hydrocyanic acid.[22] Ikeda *et al*., identified saponins (nigrumin I and II) as active compounds that showed hepatoprotective effects.[23] However, there has been no detailed biochemical study carried out on *Solanum nigrum* fruits against ethanol-induced toxicity. Keeping this in view, the present investigation on the aqueous fruit extract of *Solanum nigrum* has been undertaken to evaluate its antioxidant and antihyperlipidemic effect on ethanol-induced toxicity in rats.

## MATERIALS AND METHODS

### Chemicals

Ethanol was obtained from E. Merck, Darmstadt, Germany and E.I.D. Parry India Ltd. (Nellikuppam, Cuddalore District, South India). All other chemicals and acids were of certified analytical grade and purchased from S.D. Fine Chemicals, Mumbai or Himedia Laboratories Pvt. Ltd., Mumbai, India.

### Plant material

*Solanum nigrum* Linn (Solanaceae) fruits were collected in and around Chidambaram, Cuddalore district, Tamil Nadu, India. The herbarium of the plant was identified and authenticated by the botanist Dr. V. Venkatesalu and the voucher specimen was deposited to the Department of Botany, Annamalai University, Tamil Nadu, India.

### Preparation of aqueous fruit extract

*Solanum nigrum* fruits were washed, shade dried and finely powdered. The powder 100g, was suspended in 250 ml of water for two hours and then heated at 60-65°C for 30 minutes. The extract was collected separately and the processes were repeated thrice with the residual powder, each time collecting the extract. The collected extracts were pooled and passed through a fine cotton cloth. The filtrates were evaporated at 40-50°C in a rotavapour under reduced pressure. The dark semisolid material (yield-14%) obtained was stored at 0-4°C until use.[24] A known amount of the residual extract was suspended in distilled water and was orally administered to the animals via gastric intubation.

### Animals and diet

Adult male albino wistar rats weighing (150-170 g) were used for the study. Animals were procured from the central animal house, Department of Experimental Medicine, Raja Muthiah Medical College, Annamalai University. All the animals were acclimatized for a week under standard husbandry conditions. The animals were housed in polypropylene cages (45 × 24 × 15 cm), maintained under the temperature of 25 ± 2°C, in 12 hours light/12 hours dark conditions. The animals had free access to the standard pellet diet (Agro Corporation Private Limited, Bangalore, India), and water *ad libitum* was available to the animals throughout the experimental period, which was replenished daily. The standard pellet diet comprised of 21% protein, 5% lipids, 4% crude fiber, 8% ash, 1% calcium, 0.6% phosphorous, 3.4% glucose, 2% vitamin and 55% nitrogen-free extract (carbohydrate) and it provided a metabolizable energy of 3600 kcal/kg.

Animal handling and experimental procedures were approved by the Institutional Animal Ethics Committee, Annamalai University (Reg No: 466/160/1999/CPCSEA), and the experiments were performed in accordance with the “Guide for the care and use of laboratory animals” (NIH, 1985) and “Committee for the purpose of control and supervision on experimental animals” (CPCSEA).

### Study design

Rats were randomized into six groups of six animals each. The mode of administration for all groups was through gastric intubation. Group I control rats received 0.2 ml of gum acacia, group II rats received 20% ethanol (3.95 g/kg b.wt twice a day i.e., 7.9 g/kg/day),[25] group III rats received 20% ethanol along with SNFEt (250 mg/kg b.wt),[18] group IV rats received 20% ethanol along with the reference drug silymarin (25 mg/kg b.wt), group V rats received SNFEt alone, and group VI rats received silymarin alone. All the rats were sacrificed at the end of the experimental period of 30 days. The biochemical parameters were carried out in the circulation, liver and kidneys of all groups of animals.

At the end of the experimental period (30 days), all the rats were kept on an overnight fast and anesthetized using ketamine chloride (24 mg/kg body weight) by intramuscular injection and sacrificed by cervical decapitation between 8.00 am and 10.00 am. The blood was collected in clean dry test tubes with few drops of heparin and the plasma obtained was used for various biochemical estimations. Tissues such as the liver and kidneys were removed, the blood was cleared off and they were immediately transferred to ice-cold containers containing 0.9% NaCl. The tissues were homogenized in an appropriate buffer and used for the estimation of various biochemical parameters.

### Histopathological study

For the histopathological study, three animals from each group were perfused with physiological saline, followed by formalin (10% formaldehyde). The tissues were excised immediately and fixed in 10% formalin. The tissues were sliced and embedded in paraffin wax; 3-5 μm thick sections were cut using a microtome, dehydrated in graded alcohol, and stained with hemotoxylin and eosin. The specimens were evaluated with a light microscope. All histopathological changes were examined by the pathologist.

### Biochemical estimations

The lipid peroxidation products were estimated by measuring TBARS and were determined by Niehaus and Samuelson.[26] Nonenzymatic antioxidants such as reduced glutathione were estimated by Ellman's method,[27] ascorbic acid by Roe and Kuether's method[28] and α-tocopherol was estimated by Baker *et al*. method.[29] Lipids were extracted by the Folch method.[30] Total cholesterol, triglycerides, free fatty acids, phospholipids and HDL-cholesterol were estimated by using the respective diagnostic kits (Agappe). LDL-cholesterol was calculated as per Friedevald's formula.[31]

VLDL-Cholesterol = TG/5

LDL-Cholesterol = Serum Total cholesterol - (HDL- Cholesterol 1 VLDL-Cholesterol)

### Statistical analysis

All quantitative measurements were expressed as means ± SD for control and experimental animals. The data were analyzed using one way analysis of variance (ANOVA) on SPSS/PC (statistical package for social sciences, personal computer) and the group means were compared by Duncan's Multiple Range Test (DMRT). The results were considered statistically significant if the *P* value was less than 0.05.

## RESULTS

The body weight and organ weight of control and experimental rats are shown in Table 1. The body weight was found to be significantly reduced in the ethanol-treated rats, whereas, the liver-body weight ratio was found to be increased in animals fed with ethanol as compared to the controls. Supplementation of SNFEt (250 mg/kg b.wt) and silymarin (25 mg/kg b.wt) reversed the weight loss during the experimental period.

**Table 1:** Effect of SNFEt on body weight and liver weight to body weight ratio of control and ethanol-administered rats

Groups	Body weight (g)			
	
	Initial (0 day)	Final (30 day)	Net gain (g)	(Liver wt / body wt) × 100
Control	152.78 ± 4.31	176.50 ± 8.41^a^	23.72 ± 4.10	1.98 ± 0.62^a^
Ethanol	160.83 ± 7.02	167.00 ± 7.49^b^	6.17 ± 0.47	3.94 ± 1.78^b^
Ethanol + SNFEt (250 mg/kg b.wt)	157.11 ± 6.26	171.00 ± 5.04^c^	13.89 ± 1.22	2.28 ± 1.05^c^
Ethanol ± Silymarin (25 mg/kg b.wt)	156.21 ± 3.84	175.00 ± 5.66^c^	18.79 ± 1.82	2.17± 0.87^c^
Control SNFEt	153.63 ± 5.77	173.60 ± 7.54^ac^	19.97 ± 1.77	2.07 ± 0.68^a^
Control ± Silymarin	155.00 ± 3.51	175.00 ± 7.96^ac^	20.00 ± 4.45	2.00 ± 0.53^a^

Values are expressed as mean ± SD for six rats in each group. Values not sharing a common superscript differ significantly at *P* < 0.05 (DMRT)

Lipid peroxidation was assessed by measuring TBARS in the plasma and tissues [Table 2]. The levels of TBARS in the ethanol-intoxicated groups were significantly raised when compared to the controls (*P* < 0.05). Oral administration of SNFEt lowered the lipid peroxidation level, which was brought to near normal. Silymarin also inhibited the elevating TBARS levels on ethanol administration. No significant changes were observed in the controls and controls treated with SNFEt and silymarin.

**Table 2:** Effect of SNFEt on TBARS in the plasma and tissues of control and ethanol-administered rats

Groups	TBARS		
	
	Plasma (mmoles/dl)	Liver (mmoles/100 g wet tissue)	Kidney (mmoles/100 g wet tissue)
Control	0.18 ± 0.01^a^	0.72 ± 0.06^a^	1.35 ± 0.12^a^
Ethanol	0.41 ± 0.03^b^	2.18 ± 0.52^b^	2.78 ± 0.28^b^
Ethanol + SNFEt (250 mg/kg b.wt)	0.23 ± 0.02^c^	1.66 ± 0.08^c^	2.05 ± 0.18^c^
Ethanol + Silymarin (25 mg/kg b.wt)	0.20 ± 0.02^a^	1.47 ± 0.07^c^	1.91 ± 0.02^c^
Control + SNFEt	0.13 ± 0.01^d^	0.68 ± 0.16^a^	1.24 ± 0.17^a^
Control + Silymarin	0.18 ± 0.01^a^	0.78 ± 0.05^a^	1.36 ± 0.05^a^

Values are expressed as mean ± SD for six rats in each group. Values not sharing a common superscript differ significantly at *P* < 0.05 (DMRT)

Tables 3–5 show the concentration of nonenzymatic antioxidants such as GSH, Vitamin C and Vitamin E in the plasma, liver and kidney tissues of the control and experimental animals. The concentration of nonenzymatic antioxidants was markedly lower in the ethanol-fed group when compared to all other groups. Co-administration of SNFEt and silymarin significantly improved the antioxidant status. There were no significant changes observed in the controls and control-treated groups.

**Table 3:** Effect of SNFEt on the concentration of reduced glutathione in the plasma and tissues of control and ethanol-administered rats

Groups	Reduced glutathione		
	
	Plasma (mg/dL)	Liver (mg/100 g wet tissue)	Kidney (mg/100 g wet tissue)
Control	31.23 ± 3.51^a^	136.57 ± 9.86^a^	113.52 ± 9.14^a^
Ethanol	15.44 ± 1.36^b^	78.21 ± 6.50^b^	63.87 ± 5.57^b^
Ethanol + SNFEt (250 mg/kg b.wt)	24.31 ± 1.75^c^	95.31 ± 7.92^c^	96.41 ± 7.81^c^
Ethanol + Silymarin (25 mg/kg b.wt)	26.11 ± 2.06^cd^	108.87 ± 8.94^d^	103.62 ± 8.87^ac^
Control + SNFEt	28.43 ± 2.14^ad^	131.42 ± 9.44^a^	113.21 ± 9.05^a^
Control + Silymarin	30.20 ± 2.77^a^	134.26 ± 9.67^a^	109.43 ± 8.99^a^

Values are given as means±SD for six rats in each group. Values not sharing a common superscript differ significantly at *P* < 0.05 (DMRT)

**Table 4:** Effect of SNFEt on vitamin C in the plasma and tissues of control and ethanol-administered rats

Groups	Vitamin C		
	
	Plasma (mg/dL)	Liver (mg/100 g wet tissue)	Kidney (mg/100 g wet tissue)
Control	2.08 ± 0.25^a^	0.68 ± 0.05^a^	0.57 ± 0.05^ad^
Ethanol	1.15 ± 0.08^b^	0.21 ± 0.01^b^	0.33 ± 0.02^b^
Ethanol SNFEt (250 mg/kg b.wt)	1.66 ± 0.15^c^	0.49 ± 0.03^c^	0.50 ± 0.03^c^
Ethanol + Silymarin(25 mg/kg b.wt)	1.74 ± 0.16^c^	0.57 ± 0.01^d^	0.52 ± 0.03^cd^
Control + SNFEt	2.06 ± 0.23^a^	0.60 ± 0.04^d^	0.55 ± 0.04^acd^
Control ± Silymarin	2.10 ± 0.27^a^	0.66 ± 0.06^a^	0.59 ± 0.06^a^

Values are given as means + SD for six rats in each group. Values not sharing a common superscript differ significantly at *P* < 0.05 (DMRT)

**Table 5:** Effect of SNFEt on vitamin E in the plasma and tissues of control and ethanol-administered rats

Groups	Vitamin E		
	
	Plasma (mg/dL)	Liver (mg/100 g wet tissue)	Kidney (mg/100 g wet tissue)
Control	2.31 ± 0.05^a^	5.86 ± 0.44^a^	3.64 ± 0.46^a^
Ethanol	1.24 ± 0.14^b^	3.20 ± 0.14^b^	1.93 ± 0.77^b^
Ethanol + SNFEt (250 mg/kg b.wt)	1.92 ± 0.18^c^	4.33 ± 0.29^c^	3.11 ± 0.05^c^
Ethanol ± Silymarin(25 mg/kg b.wt)	2.08 ± 0.05^d^	4.55 ± 0.34^c^	3.38 ± 0.23^ac^
Control + SNFEt	2.47 ± 0.03^e^	5.72 ± 0.39^a^	3.59 ± 0.44^a^
Control + Silymarin	2.25 ± 0.02^a^	5.96 ± 0.48^a^	3.60 ± 0.34^a^

Values are given as means±SD for six rats in each group. Values not sharing a common superscript differ significantly at *P*< 0.05 (DMRT)

Table 6 shows the levels of lipid profile in plasma. An increased level of total cholesterol, LDL-C, VLDL-C, triglycerides, phospholipids and free fatty acids, and decreased level of HDL-C were observed in ethanol-intoxicated rats. Oral administration of SNFEt significantly improved the lipid profile levels, which were brought to near normal. The effect of SNFEt was comparable with that of the standard drug silymarin.

**Table 6:** Effect of SNFEt on the lipid profile of the plasma of control and ethanol-administered rats

Groups	Plasma (mg/dL)						
	
	Total cholesterol	HDL-C	LDL-C	VLDL-C	Triglycerides	Phospholipids	Free Fatty acids
Control	80.61 ± 6.34^a^	45.21 ± 3.98^a^	23.93 ± 1.78^a^	11.47 ± 0.75^a^	57.36 ± 3.77^a^	102.15 ± 8.50^a^	55.77 ± 3.67^a^
Ethanol	135.28 ± 8.29^b^	26.42 ± 2.30^b^	82.35 ± 4.69^b^	26.51 ± 1.81^b^	132.58 ± 9.07^b^	153.62 ± 12.58^b^	118.24 ± 8.02^b^
Ethanol + SNFEt(250 mg/kg b.wt)	110.63 ± 7.61^c^	41.73 ± 3.80^c^	51.21 ± 2.23^c^	19.05 ± 1.26^c^	95.27 ± 6.32^c^	124.75 ± 9.09^c^	94.39 ± 6.48^c^
Ethanol + Silymarin(25 mg/kg b.wt)	93.32 ± 7.78^d^	44.36 ± 2.80^ac^	32.63 ± 3.50^d^	17.43 ± 1.40^d^	87.15 ± 7.00^d^	110.83 ± 8.03^d^	92.18 ± 6.42^c^
Control + SNFEt	82.42 ± 6.56^a^	46.57 ± 3.18^a^	25.41 ± 2.60^a^	10.44 ± 0.70^a^	52.23 ± 3.50^a^	97.59 ± 5.94^a^	54.30 ± 3.51^a^
Control + Silymarin	71.29 ± 5.06^e^	45.14 ± 1.70^a^	14.73 ± 0.46^e^	11.42 ± 0.74^a^	57.12 ± 3.70^a^	105.08 ± 8.66^ad^	51.23 ± 3.34^a^

Values are given as means±SD for six rats in each group. Values not sharing a common superscript differ significantly at *P* > 0.05 (DMRT)

The data in Tables 7 and 8 depict the changes in the levels of lipid profile in the liver and kidneys of the control and experimental animals. The levels of total cholesterol, triglycerides, and free fatty acids were significantly increased, and the phospholipids were significantly decreased in ethanol-fed rats. Co-administration of SNFEt progressively improved the lipid profile toward normal when compared to silymarin.

**Table 7:** Effect of SNFEt on the lipid profile of the liver of control and ethanol-administered rats

Groups	Liver (mg/g of tissue)			
	
	Total cholesterol	Triglycerides	Phospholipids	Free Fatty acids
Control	4.68 ± 0.30^ad^	3.51 ± 0.25^a^	17.94 ± 1.46^a^	6.88 ± 0.52^a^
Ethanol	6.51 ± 0.58^b^	7.14 ± 0.63^b^	9.56 ± 0.52^b^	11.55 ± 1.01^b^
Ethanol + SNFEt (250 mg/kg b.wt)	5.40 ± 0.38^c^	4.54 ± 0.35^c^	21.38 ± 1.69^c^	9.32 ± 0.80^c^
Ethanol ± Silymarin(25 mg/kg b.wt)	5.10 ± 0.37^cd^	4.05 ± 0.30^d^	20.49 ± 1.51^cd^	8.15 ± 0.23^d^
Control ± SNFEt	4.48 ± 0.43^a^	3.31 ± 0.23^a^	17.62 ± 1.31^a^	6.33 ± 0.16^a^
Control ± Silymarin	3.88 ± 0.27^e^	3.48 ± 0.32^a^	18.44 ± 1.82^ad^	6.70 ± 0.37^a^

Values are given as means ± SD for six rats in each group. Values not sharing a common superscript differ significantly at *P* < 0.05 (DMRT)

**Table 8:** Effect of SNFEt on the lipid profile of the kidneys of control and ethanol-treated rats

Groups	Kidney (mg/g of tissue)			
	
	Total cholesterol	Triglycerides	Phospholipids	Free Fatty acids
Control	4.59 ± 0.37^a^	4.52 ± 0.35^a^	16.73 ± 1.52^ad^	3.56 ± 0.46^a^
Ethanol	6.83 ± 0.57^b^	6.74 ± 0.89^b^	7.55 ± 0.41^b^	7.72 ± 0.66^b^
Ethanol + SNFEt (250 mg/kg b.wt)	5.30 ± 0.46^c^	5.47 ± 0.54^c^	20.27 ± 2.08^c^	5.18 ± 0.27^c^
Ethanol + Silymarin(25 mg/kg b.wt)	5.02 ± 0.41^ac^	5.05 ± 0.38^ac^	18.63 ± 1.91^cd^	4.50 ± 0.40^d^
Control + SNFEt	4.81 ± 0.39^ac^	4.45 ± 0.28^a^	15.32 ± 1.28^a^	3.32 ± 0.15^a^
Control + Silymarin	4.52 ± 0.28^a^	4.50 ± 0.40^a^	16.20 ± 1.33^ad^	3.17 ± 0.06^a^

Values are given as means ± SD for six rats in each group. Values not sharing a common superscript differ significantly at *P* < 0.05 (DMRT)

### Histopathological examination

**Figure 1:**
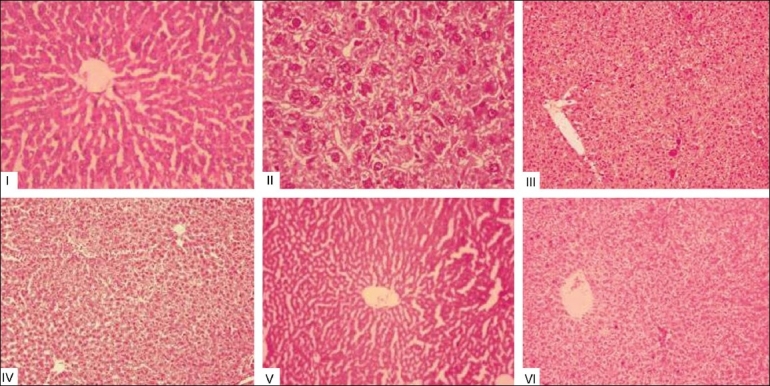
Histopathological examination of liver tissues of control and experimental animals; Group I (Control) Liver shows central vein surrounded by normal hepatocytes; Group II (Ethanol) Liver shows feathery degeneration, micro- and macrovesicular fatty changes, periportal fibrosis, and vascular congestion; Group III (Ethanol + SNFEt) liver shows normal histology with mild congestion of the central vein; Group IV (Ethanol + Silymarin) liver shows normal cells with mild inflammation in the portal triad; Group V (Control + SNFEt) liver shows central vein surrounded by normal hepatocytes; Group VI (Control + Silymarin) liver shows normal hepatocytes

Histopathological examinations of the livers of experimental rats are shown in Figure 1. Alcohol-administered rats showed lipid accumulation in large (macrovesicular) and small (microvesicular) droplets within hepatocytes and feathery generation, periportal fibrosis and vascular congestion were observed within the hepatocytes. SNFEt treatment along with alcohol showed normal histology with mild congestion of the central vein, whereas, silymarin treatment showed normal cells with mild inflammation in the portal triad. The remaining groups showed normal hepatocytes.

## DISCUSSION

In this study, it is obvious that chronic ethanol administration produced toxicity in rats, which was monitored by weight loss. Unlike other drugs, ethanol is a substantial source of energy with 7.1 kcal/g. Excessive alcohol ingestion disturbs the metabolism of most nutrients in the diet resulting in primary or secondary malnutrition. Malnutrition may be caused by either maldigestion or malabsorption and impaired utilization of nutrients,[32] which leads to significant weight loss. Supplementation of SNFEt increased the body weight to near normal, which suggests the protective effect of SNFEt against toxicity.

Oxidative stress due to the formation of free radicals was incriminated as one of the mechanisms underlying ethanol-induced toxicity. Chronic alcohol intake generates excess production of free radicals where the antioxidant defenses are impaired, which results in sequential degradation of cell membranes by a process known as lipid peroxidation. This process may destroy the integrity of the membranes both within and surrounding the cells, seriously compromising cell function.[33] Researchers have demonstrated that chronic alcohol consumption induces lipid peroxidation in rats and that the degree of lipid peroxidation is related to the extent of liver injury.[34] In agreement with these findings, our results show increased levels of lipid peroxidative markers, such as TBARS, in the circulation and tissues of alcoholic rats when compared to controls. On the other hand, treatment with SNFEt and silymarin causes a significant decline in the levels of lipid peroxidation products to near normal. This protective effect is probably based on the antioxidant activity of the extract, which reduces the oxidative damage by blocking the production of free radicals and inhibits lipid peroxidation. In line with our findings, Lin *et al*. have also reported that the *Solanum nigrum* extract inhibits the progress of lipid peroxidation on CCL_4_-administered rats.[19]

Antioxidants play a major role in protecting the cells from oxidative damage. Nonenzymatic antioxidant systems such as GSH and Vitamins C and E are considered as the second line of defense against free radicals. GSH is a major non-protein thiol, presumed to be an important endogenous defense against the peroxidative destruction of cellular membranes. In our study, the concentration of GSH was significantly reduced in alcoholic-treated rats, which was also in agreement with other reports.[35,36] This reduced level might be due to reactive oxygen intermediates generated during alcohol metabolism, which led to glutathione oxidation and lipid peroxidation. The reduced form of GSH, therefore, became readily oxidized to GSSG on interacting with the free radicals.[37] Antioxidants other than GSH might also play a role in preventing lipid peroxidation under experimental conditions.

Vitamins C and E are naturally occurring free radical scavengers.[38] Vitamin C (ascorbic acid) is an important water-soluble antioxidant in biological fluids and an essential micronutrient required for the normal metabolic functioning of the body. It is seen to react directly with superoxides,[39,40] hydroxyl radicals,[41] and singlet oxygen.[42] Vitamin C undergoes synergistic interactions with tocopheroxyl radicals, in the regeneration of α-tocopherol.[43] Decreased concentration of water-soluble antioxidants in ethanol-intoxicated rats may be due to the utilization of antioxidants to scavenge the excess amount of free radicals.[44] Vitamin E, the major lipid soluble antioxidant present in all cellular membranes, acts as a powerful terminator of lipid peroxidation.[45] Decreased levels of vitamin E are observed in the plasma and tissues of ethanol-treated rats, which may be due to the reduced concentration of Vitamin C and GSH levels, which can result in the reduced conversion of an α-tocopheroxyl radical to an α-tocopherol. Moreover many recent studies have shown that a chronic alcoholic is deficient in Vitamins C and E.[46,47]

Treatment with SNFEt significantly modulated the nonenzymatic antioxidants to near normal, which may be due to their quenching and free radical scavenging action. *Solanum nigrum* is reported to act as an effective antioxidant of major importance against diseases and degenerative processes caused by oxidative stress.[48,19] Thus, the antioxidant property of the extract may be due to the presence of a high content of polyphenolic compounds such as flavonoids, steroids, vitamin C, and β-carotene. Lee *et al*., have reported that *Solanum nigrum* glycoprotein has a strong scavenging activity against lipid peroxidation peroxyl radicals.[49] From these findings it can be inferred that SNFEt positively modulates the antioxidant status and regenerates the liver to near normal.

Lipids are a heterogenous group containing active metabolic substances that play an important role in the pathogenesis of alcoholic liver disease. Ethanol is a powerful indicator of hyperlipidemia in both animals and humans.[50] The most common lipid abnormalities during chronic alcohol consumption are known to produce hypercholesterolemia and hypertriglyceridemia.[51,52]

As a major organ of the antioxidant defense system, the liver plays a pivotal role in the regulation of lipoprotein transport in plasma and cholesterol biosynthesis. During lipoprotein transport LDL and HDL appear to be particularly important. LDL is a converted form of VLDL, rich in cholesterol and cholesterol esters, and is regarded as bad cholesterol, whereas, HDL contains relatively little cholesterol, as high levels are associated with hyperlipidemia. Ethanol-intoxicated groups showed increased levels of cholesterol in plasma and tissues, while LDL-cholesterol was remarkably increased in plasma and HDL-cholesterol was found to be reduced. The increased cholesterol during alcohol ingestion is attributed to the increased β-hydroxyl methyl glutaryl CoA (HMG CoA) reductase activity, which is the rate limiting step in cholesterol biosynthesis.[53]

Fatty liver results mainly from the accumulation of TG.[54] Increased TG levels after ethanol ingestion may be due to the increased availability of FFA, glycerophosphates, decreased TG lipase activity, and decreased fatty oxidation. These increased TG levels may lead to increased availability of FFA for esterification. Free fatty acids are the principal components present in most lipids of biological importance. The observed increase in the levels of FFA may be directly due to lipid breakdown and indirectly due to the oxidation of ethanol to acetate, which in turn forms FFA. Reports show that an increase in FFA level can increase the synthesis of other major lipids and activate NADPH- or NADH-dependent microsomal peroxidation.[55] Probably alcohol ingestion may produce the formation of esterification products of fatty acids and alcohol, which may be the mediators of end-organ damage.

Phospholipids are the vital components of a biomembrane and mainly act as regulators of membrane-bound enzymes important in determining the pathology of alcoholism.[56] The decreased phospholipid levels in the liver and kidneys may be due to the increased activity of phospholipases in these tissues. Earlier studies have demonstrated that chronic exposure to ethanol may lead to a progressive increase in membrane phospholipase A_2_ activity.[57] Hence, the alteration in the membrane composition may be the reason for the toxic defects caused by ethanol.

With the above-mentioned observation we found that supplementation of SNFEt restored the lipid levels to near normal indicating the efficacy of *Solanum nigrum*, showing antihyperlipidemic activity. It has been reported that the *Solanum nigrum* glycoprotein possesses hypolipidemic activity by increasing the antioxidant enzyme activity in normal mice. The possible reason for the lowering of the cholesterol level is that the HMG CoA reductase activity may be lost through phospholigation by cAMP-dependent protein kinase (PKA), which is activated by the *Solanum nigrum* glycoprotein.[49] Therefore, we speculate that SNFEt modulates the lipid abnormalities by inhibiting the activity of hepatic HMG-CoA reductase, thus bringing the lipid levels to normal.

## CONCLUSION

From the results, we conclude that supplementation of SNFEt exerts a significant antioxidant and antihyperlipidemic activity against ethanol-induced toxicity. This protective effect of the extract may be mainly attributed to steroidal saponins, namely nigrumin I and II, which may possess antioxidant and detoxifying effects. Therefore, a dietary intake of *Solanum nigrum* fruits supplies our body with nutrients that offer protection against numerous diseases. Further studies are warranted to elucidate the mechanisms of action and to explore its medicinal value.
